# Single domain antibody-scFv conjugate targeting amyloid β and TfR penetrates the blood–brain barrier and interacts with amyloid β

**DOI:** 10.1080/19420862.2024.2410968

**Published:** 2024-10-02

**Authors:** Rebecca Faresjö, Elisabet O. Sjöström, Tiffany Dallas, Magnus M. Berglund, Jonas Eriksson, Dag Sehlin, Stina Syvänen

**Affiliations:** a Department of Public Health and Caring Sciences, Uppsala University, Uppsala, Sweden; b Key2Brain AB, Solna, Sweden; c PET Centre, Uppsala University Hospital, Uppsala, Sweden; d Department of Medicinal Chemistry, Uppsala University, Sweden

**Keywords:** Blood-brain barrier, brain delivery, camelid antibody, fusion protein, transferrin receptor, VHH

## Abstract

Neurodegenerative diseases such as Alzheimer’s disease (AD) pose substantial challenges to patients and health-care systems, particularly in countries with aging populations. Immunotherapies, including the marketed antibodies lecanemab (Leqembi®) and donanemab (Kisunla^TM^), offer promise but face hurdles due to limited delivery across the blood–brain barrier (BBB). This limitation necessitates high doses, resulting in increased costs and a higher risk of side effects. This study explores transferrin receptor (TfR)-binding camelid single-domain antibodies (VHHs) for facilitated brain delivery. We developed and evaluated fusion proteins (FPs) combining VHHs with human IgG Fc domains or single-chain variable fragments (scFvs) of the anti-amyloid-beta (Aβ) antibody 3D6. *In vitro* assessments showed varying affinities of the FPs for TfR. *In vivo* evaluations indicated that specific VHH-Fc and VHH-scFv fusions reached significant brain concentrations, emphasizing the importance of optimal TfR binding affinities. The VHH-scFv fusions were further investigated in mouse models with Aβ pathology, showing higher retention compared to wild-type mice without Aβ pathology. Our findings suggest that these novel VHH-based FPs hold potential for therapeutic and diagnostic applications in AD, providing a strategy to overcome BBB limitations and enhance brain targeting of antibody-based treatments. Furthermore, our results suggest that a given bispecific TfR-binding fusion format has a window of “optimal” affinity where parenchymal delivery is adequate, while blood pharmacokinetics aligns with the desired application of the fusion protein.

## Introduction

Neurodegenerative diseases, such as Alzheimer’s disease (AD), are associated with an increasing number of patients, posing burdens to the families and relatives of those affected and significant societal costs. Recognized as a public health crisis, the prevalence of AD and other neurodegenerative diseases is expected to increase in the coming decades due to the aging populations of many countries. Consequently, there is an urgent need for early detection and effective therapeutic interventions to address this growing challenge.

Immunotherapy, specifically treatments based on antibodies, is currently one of the most promising treatment options for AD. In 2023, the antibody lecanemab (Leqembi®), which targets amyloid-beta (Aβ) aggregates, was approved by the US Food and Drug Administration (FDA).^1^ Additionally, several other similar antibodies are being studied in late-phase clinical trials, including antibody donanemab (Kisunla^TM^), which was FDA-approved in 2024.^2,3^ However, antibodies show restricted delivery across the blood–brain barrier (BBB). Typically, less than 0.1% of systemically administered monoclonal antibodies (mAbs) (~150 kDa) reach the brain, with the maximum brain concentration (Cmax) only reached days after administration.^4–8^ The slow and low brain delivery necessitates large doses, which is both costly and likely to produce side effects due to high antibody concentrations in the periphery.

One strategy to enhance brain delivery of antibodies involves receptor-mediated transcytosis (RMT), with the transferrin receptor 1 (TfR1, hereafter TfR) being the most widely used and clinically validated receptor for this purpose. We have established that fusing antibodies with a murine TfR-specific (mTfR) single-chain variable fragment, scFv8D3, significantly increases brain concentrations of lecanemab and other therapeutic antibodies in mice.^5,9^ These bispecific antibodies also show rapid brain delivery. Furthermore, we have demonstrated that, after radiolabeling, the bispecific mTfR-Aβ antibodies can be used as positron emission tomography (PET) radioligands to detect Aβ pathology in mouse models of AD.^10–15^ For PET, smaller-sized bispecific formats are desirable. These formats are associated with fast systemic clearance, ideally lack the crystallizable fragment (Fc) domain, which avoids immune reactions and recycling of cell-internalized antibodies. Rapid clearance may be facilitated if the bispecific antibody is small (~60 kDa or less) and thus can be excreted via the kidneys because it is below the size limit for glomerular filtration.^9,16,17^

A promising approach to create small TfR-binders for fusion to, e.g., Aβ binding antibodies or antibody fragments, involves use of the variable domain of heavy-chain antibodies derived from camelids. These single heavy chain domain fragments (VHHs), with a molecular weight of 12–15 kDa, offer high target specificity and fast blood clearance. Compared to other small antibody fragments, such as Fab (~50 kDa) or scFvs (~25 kDa), VHHs are smaller, which can allow binding to epitopes that are hidden from larger antibodies,^18^ more stable, and easily expressed recombinantly. Several studies have shown the potential of strategies to increase the BBB penetration of VHHs, or cargos thereof,^19–22^ but, to transition preclinical proteins to clinical use, they must be translatable across species. Most antibodies designed for brain delivery via TfR transport target the apical domain of TfR to avoid interfering with endogenous transfer in binding.^6,23,24^ Since the TfR apical domain is only around 70% identical between mice and humans, most TfR binders are species-specific.^21,25^ Therefore, developing novel TfR binders that specifically target the human TfR (hTfR), including further exploration of optimal binding characteristics, are valuable for clinical applications. Strategies include using knock-in mouse models expressing hTfR or developing cross-species reactive TfR binders, such as single-domain shark antibody fragments (VNARs) or camelid VHHs.^6,26–29^ However, generating cross-species reactive TfR binders with similar affinities for both mTfR and hTfR has proven challenging. Optimal binding affinity to TfR is crucial for transport efficiency: low or intermediate affinity and monovalent binding are advantageous for therapeutic dosing, while slightly higher affinity may benefit low-dose applications.^23,30,31^

In this study, we present novel VHH TfR-binders, including human-mouse cross-species reactive variants and the successful production of fusion proteins (FPs), wherein the TfR-binding VHH is fused to either an Fc-domain or the scFv of the Aβ antibody 3D6 (scFv3D6). We further describe their ability to target pathology in a mouse model of Aβ pathology, with the aim of advancing these novel TfR-binders for future therapeutic and diagnostic applications.

## Results

### *Expression and* in vitro *evaluation of fusion proteins*

Three novel TfR-binding VHHs, along with one previously published TfR-binding VHH, were produced as VHH-Fc fusion proteins (FP_Fc_). The VHHs were genetically fused to the N-terminus of both chains of a human IgG Fc domain, creating a stable IgG-like fusion protein of 75 kDa for initial evaluation. The following FPs were designed and expressed (see also Table 1): one cross-species reactive for mTfR/hTfR (FP_Fc_1), one binding only to the mTfR (FP_Fc_2), one binding only to the hTfR (FP_Fc_3), and a reference, cross-species reactive benchmark construct (FP_Fc_4), with the VHH used herein being based on a characterized sequence in patent WO 2020/144233.^26^

**Table 1.** Description of fusion proteins and their short names.

Description	Short name
VHH1-Fc	FP_Fc_1
VHH2-Fc	FP_Fc_2
VHH3-Fc	FP_Fc_3
VHH4-Fc	FP_Fc_4
scFv3D6-VHH1	FP_scFv_1A
VHH1-scFv3D6	FP_scFv_1B
scFv3D6-VHH2	FP_scFv_2A
VHH2-scFv3D6	FP_scFv_2B
scFv3D6-VHH3	FP_scFv_3A
VHH3-scFv3D6	FP_scFv_3B
scFv3D6-VHH4	FP_scFv_4A
VHH4-scFv3D6	FP_scFv_4B

Note: FP = fusion protein.

**Figure 1.**
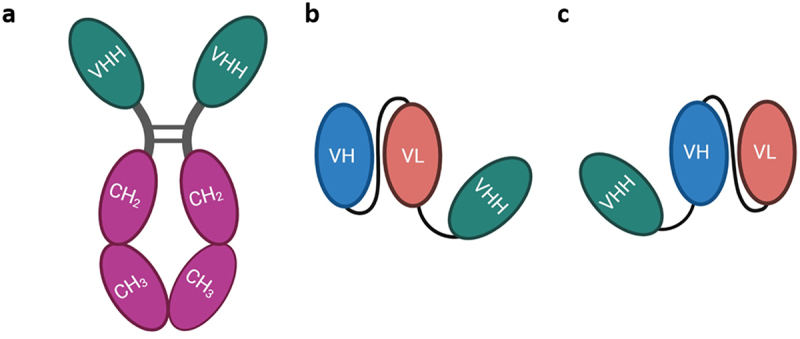
Schematic illustration of the different fusion protein formats in the paper. a) two VHH fused with human fc domain; FP_Fc._ b) single VHH fused to the C-terminus of scFv3d6; FP_scFv_A. c) single VHH fused to the N-terminus of scFv3d6; FP_scFv_B.

After initial evaluation with the four FP_Fc_, the VHHs were fused to the scFv domain of 3D6 to produce small bispecific fusion proteins (FP_scFv_) that bind monovalently to TfR and to Aβ (Figure 1).^5,32^ The variable heavy (VH) and variable light (VL) chains of scFv3D6 were connected with a 3 × (Gly_4_Ser)-linker. An identical linker was used to connect VHHs to scFv3D6. The VHHs were fused either to the C- or N-terminus of scFv3D6, denoted as “A” or “B” orientation, respectively (Table 1, Figure 1).

**Figure 2.**
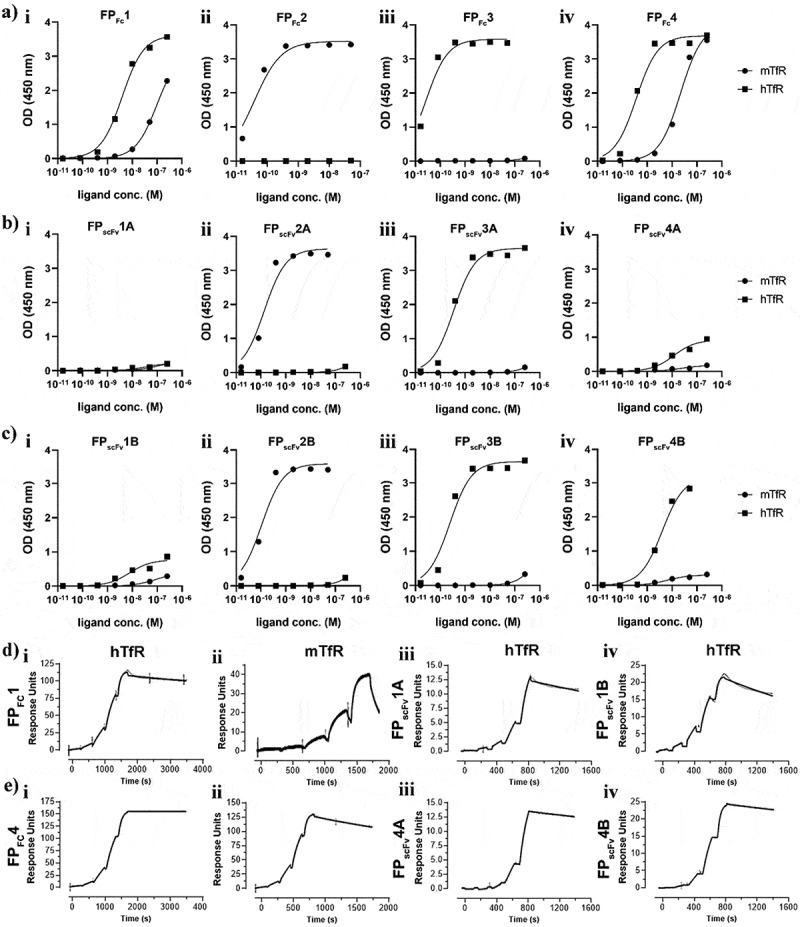
a) ELISA binding profiles for constructs in the format of fc fusions i) FP_Fc_1, ii) FP_FC_2, iii) FP_Fc_3 and iv) FP_Fc_4 to mTfR and hTfR b) ELISA binding profiles for fusions of the scFv-vhh orientation i) FP_scFv_1A, ii) FP_scFv_2A, iii) FP_scFv_3A and iv) FP_scFv_4A c) binding profiles for the fusions of the vhh-scFv orientation i) FP_scFv_1B, ii) FP_scFv_2B, iii) FP_scFv_3B and iv) FP_scFv_4B d) sensorgrams for VHH1 in the format as fc fusion (FP_Fc_1) binding to i) hTfR, ii) mTfR and in the format as fusions to anti-Aβ scFv3d6, iii) scFv-vhh orientation: FP_scFv_1A and iv) vhh-scFv orientation: FP_scFv_1B e) sensorgrams for VHH4 in the format as fc fusion (FP_Fc_4) binding to i) hTfR, ii) mTfR and in the format as fusion to anti-Aβ scFv3d6, iii) scFv-vhh orientation: FP_scFv_4A and VHH-scFv orientation: FP_scFv_4B. The receptors were immobilized by amine coupling on a CM5 chip.

The FPs were studied by enzyme-linked immunosorbent assay (ELISA) to determine their binding to mTfR and hTfR. The cross-species reactive FP_Fc_1 and FP_Fc_4, exhibited binding to both mTfR and hTfR, with higher binding to hTfR (Figure 2a i, iv). All VHH2-based constructs showed selective mTfR binding, while VHH3-based proteins bound exclusively to hTfR. The fusion of a single VHH (a monovalent TfR construct) to scFv3D6 decreased the total binding for the cross-species reactive FP_scFv_1 and FP_scFv_4, compared with that of the bivalently binding FP_Fc_1 and FP_Fc_4, with more pronounced loss of binding to mTfR compared with hTfR (Figure 2a–c i for FP1-based proteins and 2 a-c iv for FP4-based proteins). The affinity of VHH2 and VHH3 was only modestly affected by fusion to scFv3D6, as evaluated by ELISA (Figure 2b–c, ii-iii). The FP_scFv_1B and FP_scFv_4B (Figure 2c i and iv) constructs had slightly better *in vitro* binding profiles compared to FP_scFv_1A and FP_scFv_4A, respectively (Figure 2b i and iv). The binding of the scFv8D3 moiety to Aβ was not influenced by the orientation of the fusion. (Supplementary Fig S1).

The kinetic binding data for all FPs, obtained by surface plasmon resonance (SPR) analyses, are presented in Table 2. Representative sensorgrams for the human–mouse reactive FPs are shown in Figures 2d, 2e. The data agreed with the ELISA results, indicating that FP_Fc_1, as a bivalent Fc-fusion, binds to hTfR with very high avidity (0.067 nM) and to the mTfR with a *K*_D_ of 11 nM. When fused with scFv3D6, *K*_D_ values for interaction with the hTfR were 1.8 and 0.84 nM for FP_scFv_1A and FP_scFv_1B, respectively, and for the mTfR, 23 and 19 nM, respectively. The bivalent FP_Fc_4 bound to hTfR with extreme avidity (0.00053 nM, in fact below the measurable range for dissociation, (Figure 2e, 2i) and also the mTfR with very high avidity, with an equilibrium dissociation constant (*K*_D_) of 0.27 nM. When fused with scFv3D6, the *K*_D_ values were 0.70 and 0.19 nM for FP_scFv_4A and FP_scFv_4B, respectively (Table 2). FP_Fc_2, FP_scFv_2A and FP_scFv_2B did not bind hTfR and FP_Fc_3 did not bind mTfR (data not shown), thus confirming the TfR specificities of the FPs demonstrated by ELISA.

**Table 2.** SPR kinetic binding parameters. Association rate constant, k_a_; dissociation rate constant, k_d_; equilibrium dissociation constant, *K*_D._.

Construct	TfR species	k_a_ (M^−1^s^−1^)	k_d_ (s^−1^)	*K*_D_ (nM)
FP_Fc_1	human	6.51 × 10^5^	4.34 × 10^−5^	0.067
FP_Fc_1	mouse	3.54 × 10^5^	3.83 × 10^−3^	11
FP_scFv_1A	human	1.44 × 10^5^	2.59 × 10^−4^	1.8
FP_scFv_1B	human	5.35 × 10^5^	4.50 × 10^−4^	0.84
FP_scFv_1A	mouse	1.42 × 10^5^	3.28 × 10^−3^	23
FP_scFv_1B	mouse	5.17 × 10^5^	9.70 × 10^−3^	19
FP_Fc_2	mouse	1.22 × 10^5^	6.74 × 10^−5^	0.55
FP_scFv_2A	mouse	5.43 × 10^5^	1.30 × 10^−3^	4.1
FP_scFv_2B	mouse	4.89 × 10^5^	2.01 × 10^−3^	5.4
FP_Fc_3	human	8.01 × 10^5^	6.49 × 10^−5^	0.081
FP_scFv_3A	human	9.25 × 10^5^	4.77 × 10^−3^	5.2
FP_scFv_3B	human	7.77 × 10^5^	4.40 × 10^−3^	6.5
FP_Fc_4	human	5.38 × 10^5^	2.86 × 10^−7^	0.00053
FP_Fc_4	mouse	3.38 × 10^5^	9.09 × 10^−5^	0.27
FP_scFv_4A	human	1.75 × 10^5^	1.22 × 10^−4^	0.70
FP_scFv_4B	human	6.17 × 10^5^	1.17 × 10^−4^	0.19

### Ex vivo *evaluation of Fc-fusion proteins*

**Figure 3.**
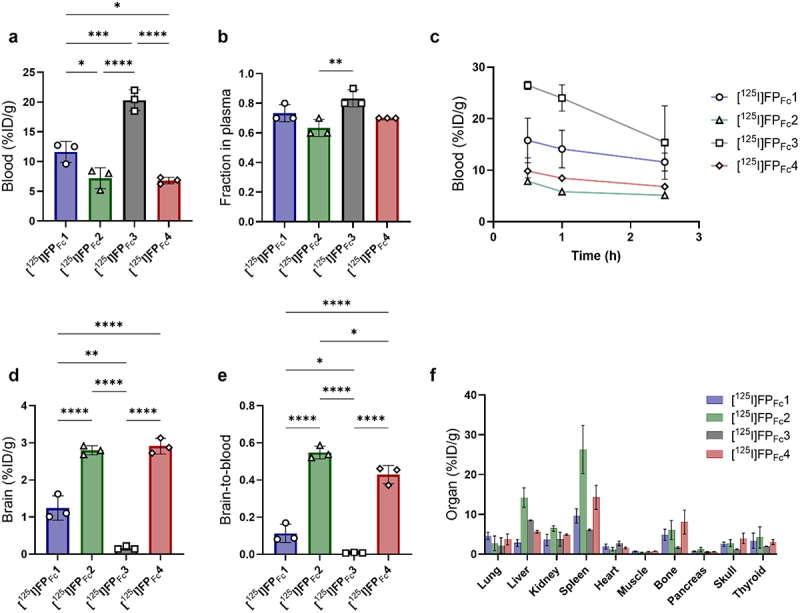
Concentrations of fc fusion proteins (FP_Fc_) 2.5 h post administration in WT mice a) blood concentration (%ID/g_blood_) b) fraction of fusion protein in plasma c) blood-time concentration curves (%ID/g_blood_) d) brain concentrations (%ID/g_brain_) e) brain-to-blood ratios and f) biodistribution to peripheral organs for the fc fusion proteins; [^125^I]FP_Fc_1, [^125^I]FP_Fc_2, [^125^I]FP_Fc_3 and [^125^I]FP_Fc_4.

Radiolabeled FP_Fc_ constructs were evaluated *ex vivo* 2.5 h after injection in wild-type (WT) mice. The hTfR binder [^125^I]FP_Fc_3, lacking affinity for the mTfR, showed the highest blood concentration at this time point. The lower-affinity cross-species reactive [^125^I]FP_Fc_1, displayed higher blood concentrations compared with the high-affinity mTfR binder [^125^I]FP_Fc_2 and the cross-species reactive [^125^I]FP_Fc_4 (Figure 3a). Given that blood cells express TfR and could potentially lower the concentration of FP available for delivery to the brain and other organs, it was also important to measure the fraction of FPs remaining in the plasma after blood cells were removed by centrifugation of the samples. The fraction present in plasma was above 60% for all FP_Fc_ constructs, but notably it was significantly higher for the hTfR binding [^125^I]FP_Fc_3 compared with the high-affinity mTfR binding [^125^I]FP_Fc_2 (Figure 3b). The blood concentration profiles showed that mTfR binders appeared to exhibit faster elimination from blood in WT mice compared with the hTfR binder [^125^I]FP_Fc_3 (Figure 3c).

The [^125^I]FP_Fc_1 displayed a total brain concentration of 1.2 ± 0.3%ID/g_brain_, which was lower than that observed for [^125^I]FP_Fc_4 and [^125^I]FP_Fc_2, which both exhibited similar brain concentrations of 2.8 ± 0.2 and 2.9 ± 0.2%ID/g_brain_, respectively (Figure 3d). The hTfR binder [^125^I]FP_Fc_3, had very low brain concentrations in WT mice, 0.17 ± 0.04%ID/g_brain_. Brain-to-blood ratios were higher for [^125^I]FP_Fc_4 and [^125^I]FP_Fc_2, compared with [^125^I]FP_Fc_1 (Figure 3e). Again, hTfR binder [^125^I]FP_Fc_3 showed a very low brain-to-blood ratio, as expected for a non-brain targeting protein. The distribution to the liver and spleen was especially high for [^125^I]FP_Fc_2, while [^125^I]FP_Fc_1 had lower distribution to these organs compared with [^125^I]FP_Fc_2 and [^125^I]FP_Fc_4, and the hTfR binder [^125^I]FP_Fc_3 (Figure 3f). The hTfR binder [^125^I]FP_Fc_3 showed limited distribution to the bone, likely due to its lack of binding to mTfR expressed by the bone marrow.

**Figure 4.**
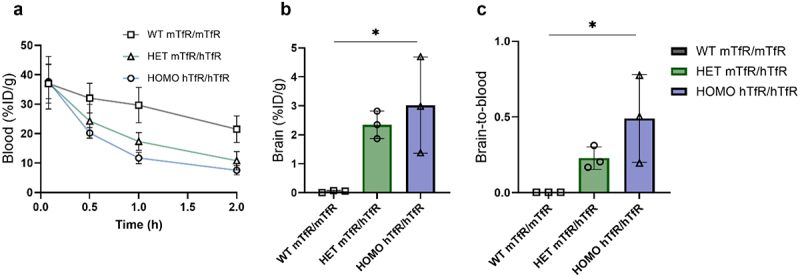
a) blood concentrations of human TfR (hTfR) binder FP_Fc_3 in WT mice homozygous for mTfR, mice heterozygous for hTfR and homozygous for hTfR. b) brain concentrations (%ID/g_brain_) and c) brain-to-blood ratios.

In contrast to what was observed in WT mice that harbor the mTfR/mTfR genotype, the hTfR binder [^125^I]FP_Fc_3 displayed the fastest elimination from blood and the highest brain delivery in mice homozygous for the hTfR (Figure 4). Thus, in homozygous hTfR mice, [^125^I]FP_Fc_3 displayed brain delivery similar to that seen with mTfR binder [^125^I]FP_Fc_2 in WT mice.

### Characterization of brain distribution of Fc-fusion proteins

**Figure 5.**
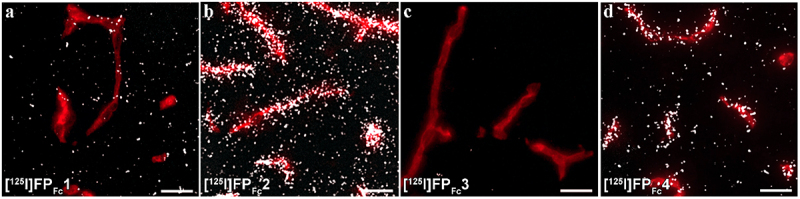
a–d) Representative images of nuclear track emulsion autoradiography illustrating the radioactivity (white) in sagittal brain sections from mice 2.5 h post-injection of [^125^I]FP_Fc_1, [^125^I]FP_Fc_2, [^125^I]FP_Fc_3 or [^125^I]FP_Fc_4 together with staining of vascular marker CD31 (red). Scale bar = 20 μm.

In WT mice, the mTfR-binding FP_Fc_ constructs [^125^I]FP_Fc_1, [^125^I]FP_Fc_2, and [^125^I]FP_Fc_4 were present in the brain capillaries (as demonstrated by CD31-positivity), in contrast to the hTfR binder [^125^I]FP_Fc_3 (Figure 5 a–d). The mTfR-specific [^125^I]FP_Fc_2 had the highest association with mouse brain capillaries (Figure 5b). Parenchymal (non-vascular) signal was also more evident for the mTfR binders compared with the hTfR-binder [^125^I]FP_Fc_3.

### Ex vivo *evaluation of scFv3D6*-*fusion proteins at 2 h post-injection*

Similar to the studies of the FP_Fc_ constructs, the FP_scFv_ constructs were evaluated at 2 h post-injection to investigate brain delivery. The FP_scFv_ constructs fused to the C-terminus of scFv3D6 (denoted with A) were compared with those fused to the N-terminus of scFv3D6 (denoted with B).

**Figure 6.**
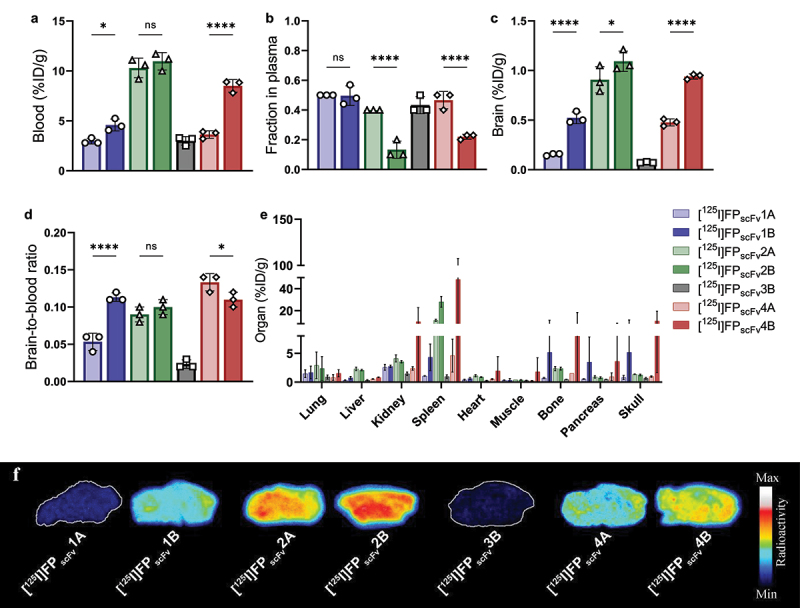
*Ex vivo* evaluation of FP_scFv_ constructs in WT mice 2 h post-injection of [^125^I]FP_scFv_1A (light blue), [^125^I]FP_scFv_1B (dark blue), [^125^I]FP_scFv_2A (light green), [^125^I]FP_scFv_2B (dark green), [^125^I]FP_scFv_3B (gray), [^125^I]FP_scFv_4A (light red) or [^125^I]FP_scFv_4B (dark red) a) terminal blood concentration (%ID/g_blood_) b) fraction of fusion protein in plasma c) brain concentration (%ID/g_brain_) d) brain-to-blood ratio e) organ biodistribution and f) representative autoradiography brain sections for^125^I-labeled FP_scFv_ constructs in WT mice.

Blood concentrations at 2 h after injections were lower for [^125^I]FP_scFv_1A and [^125^I]FP_scFv_4A compared with [^125^I]FP_scFv_1B and [^125^I]FP_scFv_4B (Figure 6a). Overall, the distribution to plasma was slightly lower compared to the respective Fc fusion and [^125^I]FP_scFv_4B and [^125^I]FP_scFv_2B displayed particularly low relative distribution to plasma (Figure 6b).

Overall, total brain concentrations at 2 h post-injection were lower for the TfR monovalent binding FP_scFv_ constructs compared to the previously studied bivalent Fc-constructs. Further, [^125^I]FP_scFv_1B displayed significantly (3-fold) higher brain concentrations than [^125^I]FP_scFv_1A, indicating that the fusion to the C-terminal end of scFv3D6 attenuated TfR binding (Figure 6c, Supplementary Table S1). The same was observed for FP_scFv_4, where [^125^I]FP_scFv_4B had 2-fold higher brain concentrations than [^125^I]FP_scFv_4A. The mTfR binder FP_scFv_2 was least affected by the orientation of the fusion, displaying similar brain concentrations for [^125^I]FP_scFv_2A and [^125^I]FP_scFv_2B. Except for [^125^I]FP_scFv_1A, all FP_scFv_ constructs directed toward mTfR, showed substantially higher brain concentrations compared with the hTfR binding [^125^I]FP_scFv_3B, indicating maintained mTfR *in vivo* binding after scFv3D6 fusion and radiolabeling.

The brain-to-blood ratio was fairly similar for all FP_scFv_ constructs, except for [^125^I]FP_scFv_1A and [^125^I]FP_scFv_3B which exhibited lower ratios due to low or no mTfR binding (Figure 6d). In line with what was observed for the FP_Fc_ constructs, biodistribution to the spleen was elevated compared to other peripheral organs for the FP_scFv_ constructs (Figure 6e). Autoradiography confirmed the previous *ex vivo* analyses of brain delivery with highest brain concentrations in [^125^I]FP_scFv_2A and [^125^I]FP_scFv_2B administered mice (Figure 6f).

Taken together, the VHH-scFv (“B-orientation”) showed significantly higher brain concentrations compared with their scFv-VHH counterparts (“A-orientation”).

### Characterization of brain distribution of scFv3d6-fusion proteins

**Figure 7.**
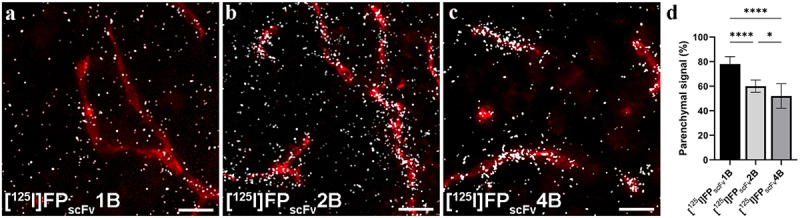
Representative images of nuclear track emulsion autoradiography illustrating the radioactivity (white) in sagittal brain sections from mice 2 h post-injection of a) [^125^I]FP_scFv_1B, b) [^125^I]FP_scFv_2B, or c) [^125^I]FP_scFv_4B, together with staining of vascular marker CD31 (red). d) quantification of the percentage of radioactive signal in parenchymal areas for [^125^I]FP_scFv_1B, [^125^I]FP_scFv_2B and [^125^I]FP_scFv_4B. Scale bar = 20 μm.

The relative distribution between capillaries and brain parenchyma was further studied with nuclear track emulsion (NTE, microautoradiography) and CD31-immunostaining for [^125^I]FP_scFv_1B, [^125^I]FP_scFv_2B and [^125^I]FP_scFv_4B (Figure 7 a–c). Although the overall brain concentrations were lower for the scFv3D6-fusions compared to the Fc-fusions, the relative levels in the capillary and parenchyma did not differ between FP_Fc_ constructs and the FP_scFv_ constructs. [^125^I]FP_scFv_1B displayed a relative distribution to the parenchyma *vs* vasculature of 78%±6%, which was higher than that seen for [^125^I]FP_scFv_2B (60 ± 5%) and [^125^I]FP_scFv_4B (52%±10%) (Figure 7d).

### Ex vivo evaluation of scFv3D6-fusion proteins at 24 h post-injection

**Figure 8.**
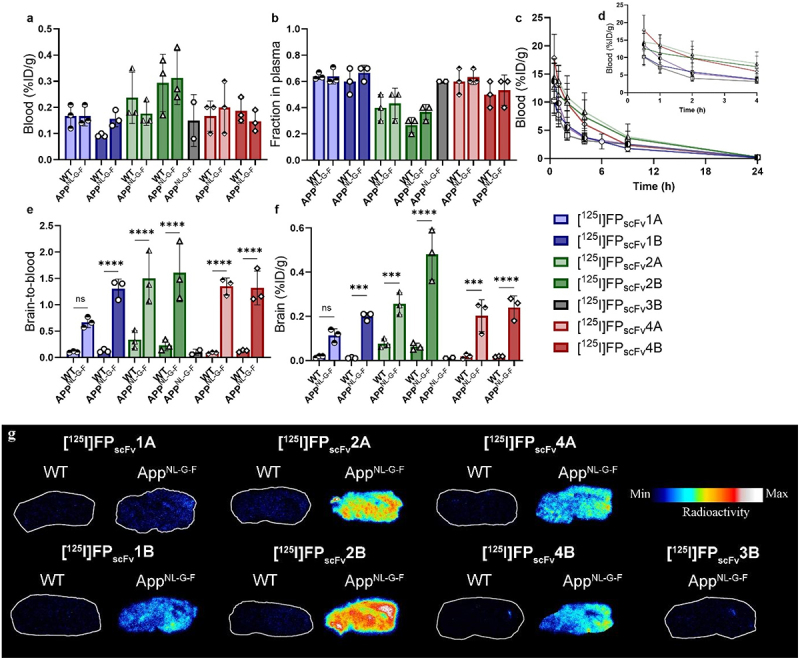
*Ex vivo* evaluation of fusion proteins (FP) in WT and *App*^*N-L-GF*^ mice 24 h post-injection of [^125^I]FP_scFv_1A, [^125^I]FP_scFv_1B, [^125^I]FP_scFv_2A, [^125^I]FP_scFv_1B, [^125^I]FP_scFv_3B, [^125^I]FP_scFv_4A or [^125^I]FP_scFv_4B a) terminal blood concentration (%ID/g_blood_) b) fraction of FP in plasma, c) blood-time concentration (%ID/g_brain_) curves during 24 h d) and during initial 4 h post injection e) brain-to-blood ratios f) brain concentrations (%ID/g_brain_) and g) representative autoradiography brain sections for^125^I-labeled FPs in WT and *App*^*N-L-GF*^ mice.

The blood and brain retention of the FP_scFv_ constructs in WT and *App*^NL-G-F^ mice were investigated at 24 h post-injection. In general, there were no significant differences in blood concentrations (Figure 8a) or fraction in plasma (Figure 8b) between WT and *App*^NL-G-F^ mice, for any of the constructs. Blood concentration curves showed that [^125^I]FPs_cFv_4B, [^125^I]FPs_cFv_2A, and [^125^I]FPs_cFv_2B appeared to display slightly slower blood elimination compared to [^125^I]FPs_cFv_1A, [^125^I]FPs_cFv_1B and [^125^I]FPs_cFv_4A (Figure 8c), corroborating results from the 2 h terminal blood samples in WT mice. All FPs, except [^125^I]FPs_cFv_1A (*p* = 0.07), displayed significantly higher brain retention in *App*^NL-G-F^ mice compared to WT mice, as indicated by both brain concentrations (Figure 8e) and brain-to-blood ratios (Figure 8f). Autoradiography of sagittal brain sections confirmed the genotype-related difference in brain accumulation, which illustrated higher retention in *App*^NL-G-F^ mice compared with WT, for [^125^I]FPs_cFv_1A, [^125^I]FPs_cFv_1B, [^125^I]FPs_cFv_2A, [^125^I]FPs_cFv_2B, [^125^I]FPs_cFv_4A and [^125^I]FPs_cFv_4B, and low retention for [^125^I]FPs_cFv_3B in *App*^NL-G-F^, indicating retention associated with genotype (Figure 8f, Supplementary Table S2). The difference in brain concentration between WT and *App*^NL-G-F^ was greatest, 17-fold, for [^125^I]FPs_cFv_1B compared to 7.6 and 11 for the other mTfR active FPs of the “B-orientation”, [^125^I]FPs_cFv_2B and [^125^I]FPs_cFv_4B, respectively (Supplementary Table S2).

### Ex vivo *evaluation of [^125^I]FP_scFv_1B and [^125^I]FP_scFv_2B at 6 h post-injection*

**Figure 9.**
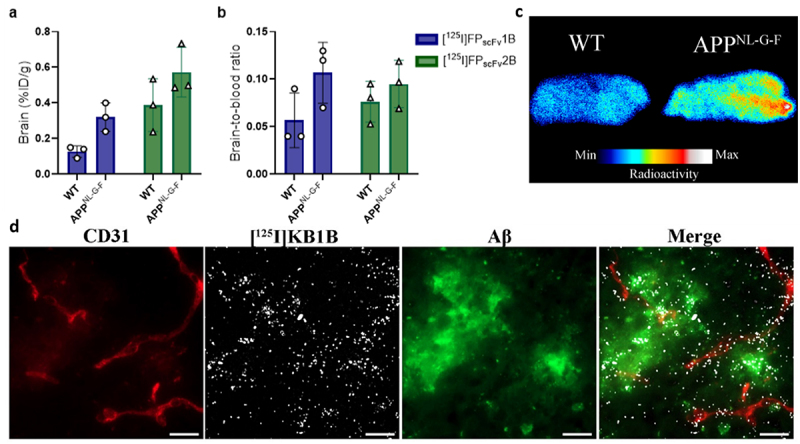
*Ex vivo* evaluation of [^125^I]FP_scFv_1B [^125^I]FP_scFv_2B in WT and *App*^*N-L-GF*^ 6 h post-injection, a) brain concentrations %ID/g_brain_ b) brain-to-blood ratio, c) representative autoradiography brain sections for [^125^I]FP_scFv_1B, showing increased retention in *App*^NL-G-F^ mice d) nuclear track emulsion of [^125^I]FP_scFv_1B in *App*^NL-G-F^ mouse brain, 6 h post-administration. Scale bar = 20 µm.

The B-orientations of the FP1 and FP2 fusions, [^125^I]FP_scFv_1B and [^125^I]FP_scFv_2B, respectively, were also studied 6 h post-injection to evaluate their potential to detect brain Aβ at an early time point, which could be relevant for a diagnostic radioligand. In line with the results at 24 h post-injection, *App*^*NL-G-F*^ mice displayed higher brain concentrations than WT mice at this time point, albeit with a smaller difference compared to 24 h; 2.5-fold and 1.5-fold for [^125^I]FP_scFv_1B and [^125^I]FP_scFv_2B, respectively (Figure 9a–b). The cross-species reactive [^125^I]FP_scFv_1B was further studies by NTE showing that [^125^I]FP_scFv_1B was associated with both Aβ pathology and to some extent also with the brain vasculature at 6 h post-administration. (Figure 9c–d).

## Discussion

Biologics are emerging as promising therapeutics and diagnostics for the brain. However, their delivery across the BBB is highly restricted, which makes targeting brain-abundant proteins such as Aβ difficult. Therefore, it is crucial to develop strategies that facilitate their transport to the brain. The TfR has been used for this purpose, serving as a shuttle for various biological molecules into the brain.^6,17,20,26,33–36^ Moreover, a bispecific antibody directed toward Aβ and TfR (trontinemab; RO7126209)^37^ has been evaluated in a Phase 1/2 study (NCT04639050). There remains, however, a need for small TfR-binders that are easy to express as fusions with the desired therapeutic or diagnostic molecules. In this study, three new TfR-binding VHH proteins, along with one previously described cross-species reactive mouse/human TfR-binding VHH, were fused to either a human Fc domain or an Aβ-specific scFv, scFv3D6, creating novel FPs. *In vitro* binding assays, including ELISA and SPR, confirmed that VHH1 and VHH4 in both their Fc and scFv fusions bound to both mTfR and hTfR. As expected, the Fc fusions, which exhibited bivalent TfR binding, showed stronger avidity toward the TfR compared to the monovalent binding scFv fusions.

*In vivo*, when the three new VHHs were fused to an Fc domain as bivalent proteins, the mTfR-specific FP_Fc_2 demonstrated higher brain distribution compared to the cross-species reactive binder FP_Fc_1. The hTfR binder FP_Fc_3 exhibited very low total brain concentration, aligning with previously described brain concentrations for a VHH devoid of TfR-mediated BBB transport.^20^ This confirmed that FP_Fc_3 was unable to cross the BBB in WT mice, but efficiently crossed the BBB in hTfR-expressing mice. In NTE, FP_Fc_2 showed the highest retention in brain capillaries compared to the other FPs, indicating that a high total brain concentration, as measured by total radioactivity, does not necessarily equate to a high concentration in the brain parenchyma. Indeed, previous studies have suggested that low to moderate TfR affinity is preferable for optimal parenchymal delivery.^31,38^ These *in vivo* results were largely consistent with the measured *in vitro* affinities, showing that FP_Fc_2 had a higher affinity toward mTfR than FP_Fc_1, and that the selective hTfR binder, FP_Fc_3, showed no affinity toward mTfR but high affinity toward hTfR.

All VHH proteins were also fused to the Aβ-binding scFv3D6. In line with initial studies of the Fc fusions, high mTfR affinity correlated with high total brain concentrations of scFv3D6 fusions at 2 h post-administration, as well as a higher fraction found in brain capillaries as measured by NTE. However, total brain concentrations were generally lower for FP_scFv_ constructs compared to FP_Fc_ constructs, likely due to reduced avidity for TfR in the monovalent format and potentially lower blood concentrations for scFv fusions compared to the larger Fc fusions. It appeared that VHH fusion to the N-terminus of scFv3D6 (“B-orientation”) resulted in higher brain concentrations compared with fusion to the C-terminus (“A-orientation”), a finding particularly notable for the cross-species reactive FP_scFv_1B. While FP_scFv_1B showed somewhat lower brain concentrations than FP_scFv_4B, it demonstrated greater relative distribution to the parenchyma and was less associated with capillaries at 2 h. Lower TfR affinity of VHH1-based fusions, compared to others, might facilitate dissociation from TfR within the endosomes of brain endothelial cells, allowing for more efficient transcytosis.^31^ *In vitro*, FP_scFv_1B and FP_scFv_4B had similar association rate constants to hTfR, but FP_scFv_1B had a nearly 4-fold higher dissociation rate constant (k_d_) and a 4-fold higher total K_D_ compared to FP_scFv_4B in SPR measurements, indicating that the lower affinity may result from faster dissociation from the receptor.

The B-orientation variants, especially FP_scFv_1B and FP_scFv_4B, showed a greater difference between mice with and without Aβ pathology compared to their A-orientation variants. This characteristic is crucial for potential future applications as diagnostic radioligands. Therefore, two N-terminal fusions, FP_scFv_1B and FP_scFv_2B, were also studied 6 h post-injection, with FP_scFv_1B showing a somewhat larger difference between AD and WT mice, likely due to faster clearance of the unbound construct from the blood and brain.

Thus, for both Fc and scFv fusions, the total brain concentration was highly correlated with affinity toward mTfR; high mTfR affinity resulted in high total brain concentration. However, high mTfR affinity also resulted in a higher fraction of the fusion protein being associated with brain capillaries. In blood, TfR affinity affected elimination, likely due to TfR-mediated clearance in the periphery.^39^ However, the impact differed for Fc and scFv fusions. For Fc fusions, high mTfR affinity was associated with fast elimination, while for scFv fusions, high mTfR affinity was associated with slower elimination. This apparent contradiction can be explained by the different molecular sizes of the Fc and scFv fusions. Smaller scFv fusions inherently clear faster. When fused to TfR binders, higher affinity increases their blood residence time. For larger Fc fusions, where the Fc domain itself prolongs residence time in the blood,^40^ TfR-mediated clearance increases elimination. Thus, high TfR affinity promotes elimination. This phenomenon has also been demonstrated with full-sized IgG antibodies, which show faster elimination when fused to high-affinity TfR binders.^5,36,41^ Another factor that may influence the “optimal” TfR affinity for brain delivery is the nature of the cargo itself. It is important that the affinity for TfR is lower than the cargo’s affinity for the target protein within the brain, such as Aβ in the case of the scFv fusions used in this study. Otherwise, there is a risk that the FPs may bind to TfR on neurons or other cells instead of targeting the intended protein.^17^

We did not measure TfR expression levels in this study. In theory, the amount of TfR could potentially influence both the delivery and the brain retention of the FPs. We have observed increased brain delivery of TfR-targeted bispecific FPs along with increased protein levels of TfR in young mice compared to old.^42^ Thus, we used aged-matched AD and WT animals for the 6 h and 24 h brain retention studies. There are conflicting reports on how Aβ pathology may influence the expression of TfR. One study reported no changes in the 3×Tg-AD mouse model or in human brain samples.^43^ However, another study reported no changes in TfR levels measured in isolated microvessels, but increased expression of TfR in cortical samples from the 5×FAD mouse model.^44^ In our own previous studies, which align with the report of no changes in TfR expressed at the BBB, we did not observe any difference in the initial brain delivery between AD and WT mice.^42^ However, in line with the reports of increased TfR in whole cortical samples that include parenchymal cells, we cannot completely rule out that a fraction of the retention of the scFv fusions at later time points may arise from binding to TfR in parenchymal cells, such as neurons or astrocytes. Yet, this contribution to total brain concentrations is likely to be small, as studies with bispecific antibodies directed toward TfR and a brain irrelevant target do not accumulate more in AD mice than in WT mice.^12,45^

In summary, we have produced and evaluated novel VHH TfR binding fusion proteins, including human-mouse TfR cross-species reactive binders successfully fused with an Aβ-binder, scFv3D6. This enabled transport across the BBB and interaction with Aβ pathology. Although high affinity leads to higher association with brain tissue, it also results in increased retention in brain capillaries and a lower fraction of the fusion protein in plasma. These processes likely reduce parenchymal distribution, indicating that lower affinity may be desirable for brain delivery. Additionally, low capillary retention and fast clearance from blood, as observed for FP_scFv_1B, reduce background radioactivity, which is important for a diagnostic radioligand. Therefore, it is likely that for a given bispecific TfR-binding fusion format, an optimal affinity window exists where parenchymal delivery is adequate, while blood pharmacokinetics align.

## Materials and methods

### Production of fusion proteins

The four FP_Fc_ were expressed in Expi293^TM^ cells at Thermo Fisher Scientific, using a murine kappa IgG light-chain signal peptide and purified using standard protein A-based systems.

The FP_scFv_ were expressed transiently in Chinese hamster ovary (CHO) cells (TurboCHO^TM^) by Genscript Corp. A 6 × Histidine C-terminal tag enabled HisTag-based protein purification. The orientation and short names of the FP_scFv_ are listed in Table 1.

### In vitro *characterization*

The binding of FPs to mTfR and hTfR was measured with indirect ELISA. Briefly, 96-well plates (Corning Inc.) were coated with 2 µg/ml of mTfR or hTfR (soluble ectodomains with N-terminal 6 × Histidine tags produced transiently in CHO cells), diluted in phosphate-buffered saline (PBS) and incubated at 4°C overnight. Blocking with 1% bovine serum albumin (BSA) in PBS was performed the following day for 2 h, after which the FPs were serially diluted (1:5) on the plate starting at 250 nM and incubated overnight at 4°C. The plates were incubated for 1 h with horseradish peroxidase (HRP) conjugated anti-VHH antibody (GenScript), and the plates were subsequently developed with K Blue Aqueous TMB substrate (Neogen Corp., Lexington, KY, USA). The reaction was stopped after 10 min with 1 M H_2_SO_4_, and optical density at 450 nm was measured using a spectrophotometer. Fusion antibody and secondary antibody sample dilutions were made in ELISA incubation buffer (PBS, 0.1% BSA, 0.05% Tween-20®).

The binding of FPs to hTfR and mTfR was also assessed by SPR in single-cycle kinetics mode using a Biacore 8K instrument (Cytiva). For each construct, 5 nM of FP_Fc_, diluted in assay buffer (PBS supplemented with 0.05% Tween-20) were loaded on a protein A chip (Cytiva) for 60 s at 10 µL/min followed by exposure to increasing concentration of receptor. Human TfR binding was assessed at a concentration range from 0.078 to 20 nM (five concentrations in four-fold increments) and the mTfR from 0.16 to 40 nM. Contact time for each concentration was 300 s at 30 µL/min and dissociation was monitored for 1800 s. After each full five-concentration run, the surface was restored in a single regeneration step by injection of 10 mm glycine at pH 2.0 for 30 s at 30 µL/min.

For the FP_scFv_ constructs, the receptors hTfR and mTfR were immobilized to a CM5 chip (Cytiva) by amine-coupling using standard methods. FP_scFv_1A/B, FP_scFv_2A/B, and FP_scFv_3A/B were tested to the hTfR at a concentration range of 0.25 to 64 nM (five concentrations in four-fold increments), whereas FP_scFv_1A/B was tested at a concentration range from 0.08 to 50 nM (five concentrations in five-fold increments). FP_scFv_1A/B and FP_scFv_2A/B were also assessed at the mTfR using the same conditions, but at a concentration range from 0.5 to 128 nM (five concentrations in four-fold increments). The contact time for each concentration was 120 s at 30 µL/min, and dissociation was monitored for 600 s. After each full five-concentration run, the surface was restored in a single regeneration step by injection of 500 mm MgCl_2_ for 30 s at 30 µL/min.

The data with respect to the association rate constant (k_a_), dissociation rate constant (k_d_) and equilibrium dissociation constant (*K*_D_) was evaluated using Biacore Insight Evaluation package (Cytiva) applying a Langmuir 1:1 model.

### Radiochemistry

For *in vivo* evaluation FPs were radiolabeled with iodine-125 (^125^I) using the chloramine T method as previously described.^42,46^ Briefly, 15–80 µg of FP was mixed with 260 ± 26 kBq/µg for FP_Fc_ or 61 ± 2.4 kBq/µg for FP_scFv_, of stock^125^I (PerkinElmer Inc, Waltham, MA, USA) and 5 µg of Chloramine T (Sigma Aldrich) in PBS. The reaction (110 µL) was allowed to incubate for 90 s before it was quenched with 10 µg of Na-metabisulfite (Sigma-Aldrich). The radiolabeled sample was immediately purified with a Zeba-column (ThermoFisher) of 7 kDa cutoff.

### Animals

Experiments to assess brain delivery of the FP_Fc_s and FP_scFv_s were carried out in 3-month-old male WT C57/BL6 mice (*n* = 33). The hTfR binding FP_Fc_3 was also studied in mice with a chimeric ectodomain of hTfR generated by Taconic Biosciences GmbH, in which the endogenous *Tfrc* gene was partially humanized so that the engineered mice produced a chimeric TFRC protein containing the human TFRC extracellular domain, a genotype previously described in literature.^29^ Mice were used as both heterozygotes, homozygotes, and WT littermates (*n* = 3 per genotype). The pharmacokinetics and brain retention of FP_scFv_s were studied in 13–15 months old *App*^NL-G-F^ mice, expressing amyloid precursor protein (APP) gene with a humanized Aβ sequence and with the Swedish (KM670/671NL), Arctic (E693G), and Iberian (I716F) mutations, and in aged-matched WT mice. The *App*^NL-G-F^ model is a knock-in model that develops plaque pathology at the age of 2–3 months.^47^ Both males (*n* = 25) and females (*n* = 26) were used in the FP_scFv_ experiments. All animals used in the study are given in Table 3.

**Table 3.** Fusion proteins, VHH reactivity, and the number of mice injected with respective FP in the study.

Fusion Protein	VHH-Reactivity	WT 3 months	WT/HET/HOMO* 2-3 months	WT 13-15 months	*App*^NL-G-F^ 13-15 months
FP_Fc_1	mTfR/hTfR	3			
FP_Fc_2	mTfR	3			
FP_Fc_3	hTfR	3	3/3/3		
FP_Fc_4	mTfR/hTfR	3			
FP_scFv_1A	mTfR/hTfR	3		3	3
FP_scFv_1B	mTfR/hTfR	3		6	6
FP_scFv_2A	mTfR	3		3	3
FP_scFv_2B	mTfR	3		6	6
FP_scFv_3B	hTfR	3			3
FP_scFv_4A	mTfR/hTfR	3		3	3
FP_scFv_4B	mTfR/hTfR	3		3	3

Note: *Mice from breeding of hTfR expressing mice.

Animals were housed in an animal facility at Uppsala University, with *ad libitum* access to food and water. All experimental procedures described were approved by the Uppsala County Animal Ethics board (5.8.18–20401/20), according to regulations of the Swedish Animal Welfare Agency, and complied with the European Communities Council Directive of 22 September 2010 (2010/63/EU).

### In vivo *brain delivery and retention*

Immediately following radiolabeling, FP_Fc_ (5 nmol/kg; 3.4 MBq/nmol) or FP_scFv_ (5 nmol/kg; 6.8 MBq/nmol) were administered as an intravenous injection to mice via the tail vein. To investigate brain targeting, mice were euthanized at 2.5 h post-injection by sampling blood from the heart before transcardial perfusion with 40 mL NaCl (room temperature) for 2.5 min. The brain and major organs were isolated. Blood was separated into plasma and blood-cell pellet by centrifugation at 10 000 g for 5 min. The brain was separated into the left and right hemispheres. The cerebellum was removed from the left hemisphere, and the remaining tissue of the left hemisphere is hereafter referred to as “brain”. Radioactivity was then measured in blood, plasma, blood cell pellet, the right hemisphere, brain, cerebellum, and major organs using a γ-counter (2480 Wizard^TM^, Wallac Oy PerkinElmer, Turku, Finland). To investigate brain retention and pharmacokinetics in blood, both WT and *App*^NL-G-F^ mice were administered with the radiolabeled FP. Blood samples (8 μL) were obtained from the tail vein at 5 min, 0.5 h, 1 h, 2 h, 4 h, and, if not euthanized at 6 h, at 9 h post injection. At 6 h or 24 h, mice were euthanized, and samples were collected according to the same procedure as described above.

### Ex vivo *autoradiography*

To visualize the spatial distribution of the radiolabeled FPs in the brain tissue, isolated right hemispheres isolated were sectioned using a Cryostar N×70 (ThermoFisher). Sagittal cryosections (20 μm) were placed in an X-ray cassette along with standards of known^125^I-radioactivity and exposed to a phosphor imaging screen (MS, MultiSensitive, PerkinElmer, Downers grove, IL, USA). The radioactive samples were exposed for seven days and were then scanned in a Cyclone Plus Imager system (Perkin Elmer), at 600 dpi. The resulting digital images were converted to a false color scale (Royal) with ImageJ and normalized to the standards.

### Immunostaining with CD31 and nuclear track emulsion

To investigate the FP relative distribution between vasculature and brain parenchyma, sagittal cryosections of 20 µm were prepared from the right-brain hemisphere of mice injected with the^125^I-labeled FP_FC_ or FP_scFv_ constructs. The sections were fixed for 10 min in ice-cold methanol, washed with PBS, and blocked with 5% normal goat serum. The sections were incubated with PBS 0.1% Tween-20 for 5 min before overnight incubation with primary antibodies rat-anti-mouse CD31 (BD, #553370), and rabbit-anti-Aβ42 (Agrisera, custom production) at 4°C with slow shaking. The following day, sections were washed with PBS and incubated with goat-anti-rat (Alexa 647) and goat-anti-rabbit (Alexa 488) secondary antibodies. The sections were stored in PBS until the NTE procedure (described below) was performed on the same day.

NTE was carried out according to previously published procedures.^4,42^ All processes were performed under darkroom conditions with a safelight according to the manufacturer’s instructions. Ilford K5 emulsion was melted in a 40°C water bath and prepared as a 50% solution in ultrapure water. The brain sections were submerged in the emulsion for 10 s, followed by air-drying in RT for 2 h. The sections were stored at 4°C, in a light-sealed box for 4 weeks. The development was done according to the manufacturer’s instructions. The sections with immunostaining and developed emulsion were visualized with a Zeiss Observer Z.1 microscope using ZEN software (Carl Zeiss Microimaging GmbH, Jena, Germany). The brightfield channel was inverted to show the emulsion grains as white puncta, instead of black, together with the CD31 staining in the red 647-channel.

Quantification of the relative parenchymal vs capillary signal (%Parenchyma) in the NTE images was done using randomly distributed cortical images (*n* = 20 per animal). Images were taken from one selected animal per group displaying the median brain concentrations. The quantification was performed with a standardized macro in Fiji (ImageJ) as previously described.^9^

### Statistical analyses

All data presented here are shown as mean ± SD. Shapiro-Wilk test and diagnostic plots were used to evaluate the normal distribution of the data. The ELISA data was fitted using saturation binding curves. One-way or two-way ANOVA followed by Bonferroni *post hoc* analyses were applied to correct for multiple comparisons. Statistically significant differences were defined as: *p*-value <0.05 (^*^), *p*-value <0.01(^**^), *p*-value <0.001 (^***^), *p*-value <0.0001 (^****^). Graphs and statistical analyses were performed in GraphPad Prism version 9.4.1 for Windows (GraphPad Software, San Diego, California, USA).

## Supplementary Material

Supplemental Material
