# Supplementary material for: Single domain antibody-scFv conjugate targeting amyloid β and TfR penetrates the blood–brain barrier and interacts with amyloid β
Source: MAbs. 2024 Oct 2;16(1):2410968. doi: 10.1080/19420862.2024.2410968 (PMC11451328; doi:10.1080/19420862.2024.2410968)
Supplement: Supplemental Material [file KMAB_A_2410968_SM8005.docx]

Supplementary files

**Supplementary Table 1:** Brain concentrations 2 h post-injection of the scFv3D6 based fusion proteins. Mean ± SD.

| *Protein* | *%ID/g_brain_* | *Protein* | *%ID/g_brain_* |
| --- | --- | --- | --- |
| [^125^I]FP_scFv_1A | 0.15±0.01 | **[^125^I]FP_scFv_1B** | 0.52±0.05 |
| [^125^I]FP_scFv_2A | 0.91±0.13 | **[^125^I]FP_scFv_2B** | 1.09±0.10 |
|  |  | **[^125^I]FP_scFv_3B** | 0.07±0.01 |
| [^125^I]FP_scFv_4A | 0.47±0.04 | **[^125^I]FP_scFv_4B** | 0.94±0.03 |

**Supplementary Table 2:** Brain concentrations 24 h post-injection of the scFv3D6 fusion proteins. Mean ± SD.

| Protein | WT  (%ID/g brain) | *App^NL-G-F^*  (%ID/g brain) | Fold difference  WT vs  *App^NL-G-F^* | Fold difference to control  KB3B *App^NL-G-F^* |
| --- | --- | --- | --- | --- |
| [^125^I]FP_scFv_1A | 0.02±0.01 | 0.11±0.02 | 6.5 | 7.0 |
| [^125^I]FP_scFv_1B | 0.01±0.01 | 0.20±0.01 | 17 | 18 |
| [^125^I]FP_scFv_2A | 0.08±0.02 | 0.26±0.04 | 3.3 | 18 |
| [^125^I]FP_scFv_2B | 0.06±0.01 | 0.48±0.09 | 7.6 | 31 |
| [^125^I]FP_scFv_3B | n/a | 0.01±0.01 | n/a | n/a |
| [^125^I]FP_scFv_4A | 0.02±0.01 | 0.20±0.01 | 11 | 10 |
| [^125^I] FP_scFv_4B | 0.02±0.01 | 0.24±0.04 | 13 | 16 |


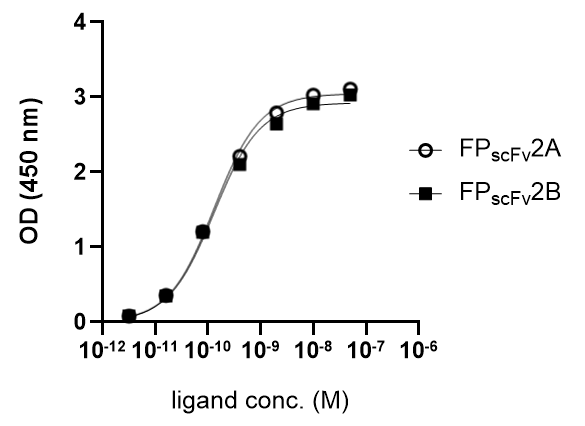


**Supplementary Fig 1:** The binding to Aβ was the same for the A- and B-orientations. Data shown for the FP_scFv_2 proteins
